# Potential use of veno-arterial extracorporeal membrane oxygenation for cardiogenic shock refractory to mechanical assist devices: baseline physiology and mortality data

**DOI:** 10.1186/cc13357

**Published:** 2014-03-17

**Authors:** C Vimalanathan, N Barrett, N Ioannou, C Langrish, C Meadows, G Salt, G Glover

**Affiliations:** 1 Guy's & St Thomas' NHS Trust, London, UK

## Introduction

Mortality from cardiogenic shock remains high [1] and, despite a physiological rationale, intra-aortic balloon counterpulsation (IABP) has recently been shown to be ineffective in reducing mortality [2],[3]. Veno-arterial extracorporeal membrane oxygenation (V-A ECMO) may offer a survival advantage over IABP. The objective of this study was to describe the characteristics and outcomes of patients supported with IABP or Impella and to identify the characteristics of patients who die, despite mechanical assistance, for whom a proposed V-A ECMO programme may be beneficial.

## Methods

A retrospective cohort study in a 30-bed, medical-surgical ICU. All adult patients supported with IABP or Impella over 2 years to March 2013 were identified and data were extracted by case-note review. Subgroup analysis was carried out for patients aged ≤65 and for those who fulfilled the modified Melbourne criteria for V-A ECMO [4]. Data collected included demographic data, physiology and organ support at baseline and at 6, 12, and 24 hours, ICU and hospital outcomes and cause of death. Comparisons between survivors and nonsurvivors were made with *t *test/chi-squared tests as appropriate.

## Results

A total of 129 patients were identified: 78% were male, mean age was 70 years (SD ±11.8), mean APACHE II score was 20 (±5) and ICU mortality was 44%. Comparing survivors with nonsurvivors the only statistically significant difference was metabolic acidosis (-6.8 ± 5.3 vs. -10.9 ± 7.0 mEq/l; *P *< 0.05). Heart rate, mean arterial pressure, lactate, central venous oxygen saturation, cardiac index, arterial blood pH and mechanical ventilation failed to show a significant difference. Eleven of these patients would have fulfilled the proposed criteria for V-A ECMO, with an ICU mortality of 36%.

## Conclusion

Only metabolic acidosis was associated with mortality in patients supported with mechanical assist devices. Our data do not allow discrimination of survivors from nonsurvivors. Patients who fulfilled the proposed criteria for V-A ECMO showed a similar mortality to a recent series treated with V-A ECMO [4]. The proposed criteria do not identify a cohort, in this population, that would expect a mortality benefit from V-A ECMO.

## References

[B1] Jeger RV Ann Intern Med 2008 149 618 626 10.7326/0003-4819-149-9-200811040-00005 18981487

[B2] Thiele H N Engl J Med 2012 367 1287 1296 10.1056/NEJMoa1208410 22920912

[B3] Werdan K Eur Heart J 2013 in press

[B4] Aubron C Crit Care 2013 17 R73 10.1186/cc12681 23594433 PMC4056036

